# Microtubule dependent sorting of actin-binding proteins in mitosis

**DOI:** 10.1038/s41598-024-61967-7

**Published:** 2024-05-16

**Authors:** Jana Prassler, Mary Ecke, Günther Gerisch

**Affiliations:** Max Planck Institute of Biochemistry, Am Klopferspitz 18, 82152 Martinsried, Germany

**Keywords:** Actin nucleation, Arp2/3 complex, Cytokinesis, Formin, Microtubules, Biological techniques, Cell biology

## Abstract

The patterns of Formin B and of the Arp2/3 complex formed during mitosis were studied in a mutant of *Dictyostelium discoideum* that produces multinucleate cells, which divide by the ingression of unilateral cleavage furrows. During cytokinesis the cells of this mutant remain spread on a glass surface where they generate a planar pattern based on the sorting-out of actin-binding proteins. During anaphase, Formin B and Arp2/3 became localized to the regions of microtubule asters around the centrosomes; Formin B in particular in the form of round, quite uniformly covered areas. These areas have been shown to be depleted of myosin II and the actin-filament crosslinker cortexillin, and to be avoided by cleavage furrows on their path into the cell.

## Introduction

Cytokinesis involves the cross-talk between the microtubule and actin systems to result in division of a mitotic cell after segregation of the chromosomes (for an overview see Burgess and Chang)^1^. Typically, single cells divide by a cleavage furrow that separates two daughter cells, each with one set of chromosomes. Here we study cytokinesis under the special conditions of large multinucleate cells that form unilateral cleavage furrows. In continuation of prior work^2,3^ cells of a Septase—mutant (SepA-null) of *Dictyostelium discoideum* were used, in which a serine-threonine kinase has been inactivated that affects the cytoskeleton. These mutant cells did not round up during mitosis and became spontaneously multinucleate; nevertheless, they formed unilateral cleavage furrows, although with a delay and less efficiently in comparison to multinucleate wild-type cells.

Unilateral furrows were initially observed in multinucleate wild-type AX2 cells of *Dictyostelium discoideum* generated by electric-pulse induced cell fusion^4^. In the multinucleate Septase-null cells, myosin II and the actin-crosslinker cortexillin formed planar patterns on the substrate-attached cell surface. Determinants of this pattern were microtubule asters that surrounded the separated centrosomes at a late anaphase stage; at the aster regions both myosin II and cortexillin were depleted^3^. Essentially, the 3-dimensional organization of a wild-type cell in cytokinesis is converted into a 2-dimensional pattern in SepA-null cells.

We used the large multinucleate cells of the Septase-null mutant to relate Formin B (ForB) and Arp2/3 to the pattern formed by myosin-II and cortexillin, two proteins that normally accumulate in the cleavage furrow^5–7^. In contrast, Arp2/3 localizes to the polar regions of the dividing cells^8^, and in interphase cells ForB co-localizes with Arp2/3 in actin-wave patterns^9^. We found that during anaphase, ForB and Arp2/3 transiently accumulate in the regions of microtubule asters just where myosin II and cortexillin are depleted.

## Results

### Localization of Formin B to microtubule asters

**Figure 1:**
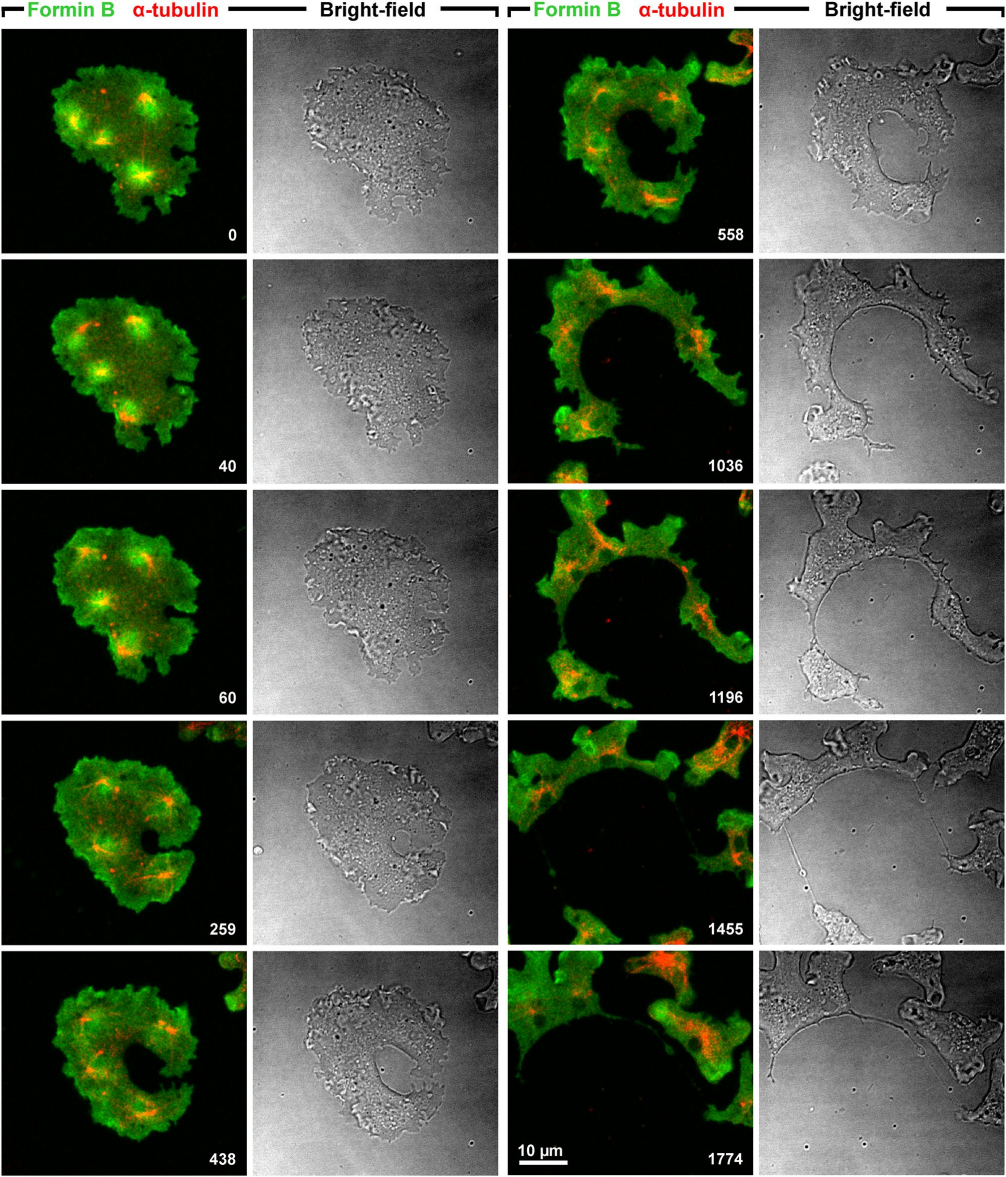
Ingression of a single unilateral cleavage furrow into a multinucleate Septase-null cell. The cell expressed GFP-ForBΔDAD (green) and RFP-α-tubulin (red). Time is indicated in seconds after the first frame. The sequence begins with the anaphase stage of elongated spindles, ForB forming bright halos around the centrosomes. During ingression of the cleavage furrow from the right cell border, ForB distributes to protrusions of the cell (438-s to 558-s frames). Later on, the cell is cleaved into mononucleate and binucleate pieces. This figure is related to Supplementary Video S1.

The localization of ForB relative to the microtubule system is illustrated in two multinucleate cells labeled with a ΔDAD construct that lacks the auto-inhibitory DAD domain as described by Körber et al.^10^. The cell shown in Fig. 1 and Supplementary Video S1 was initially cleaved by a single unilateral furrow. At the beginning of furrow ingression, ForB formed bright halos around the centrosomes (0-s to 60-s frames). This co-localization of ForB with microtubule asters was consistently found in 46 cells at all their 298 centrosomes. At late stages of furrow progression this location became lost, while the accumulation of ForB in protrusions was retained during further division and within the daughter cells that returned to the interphase stage (1036-s and following frames).

**Figure 2:**
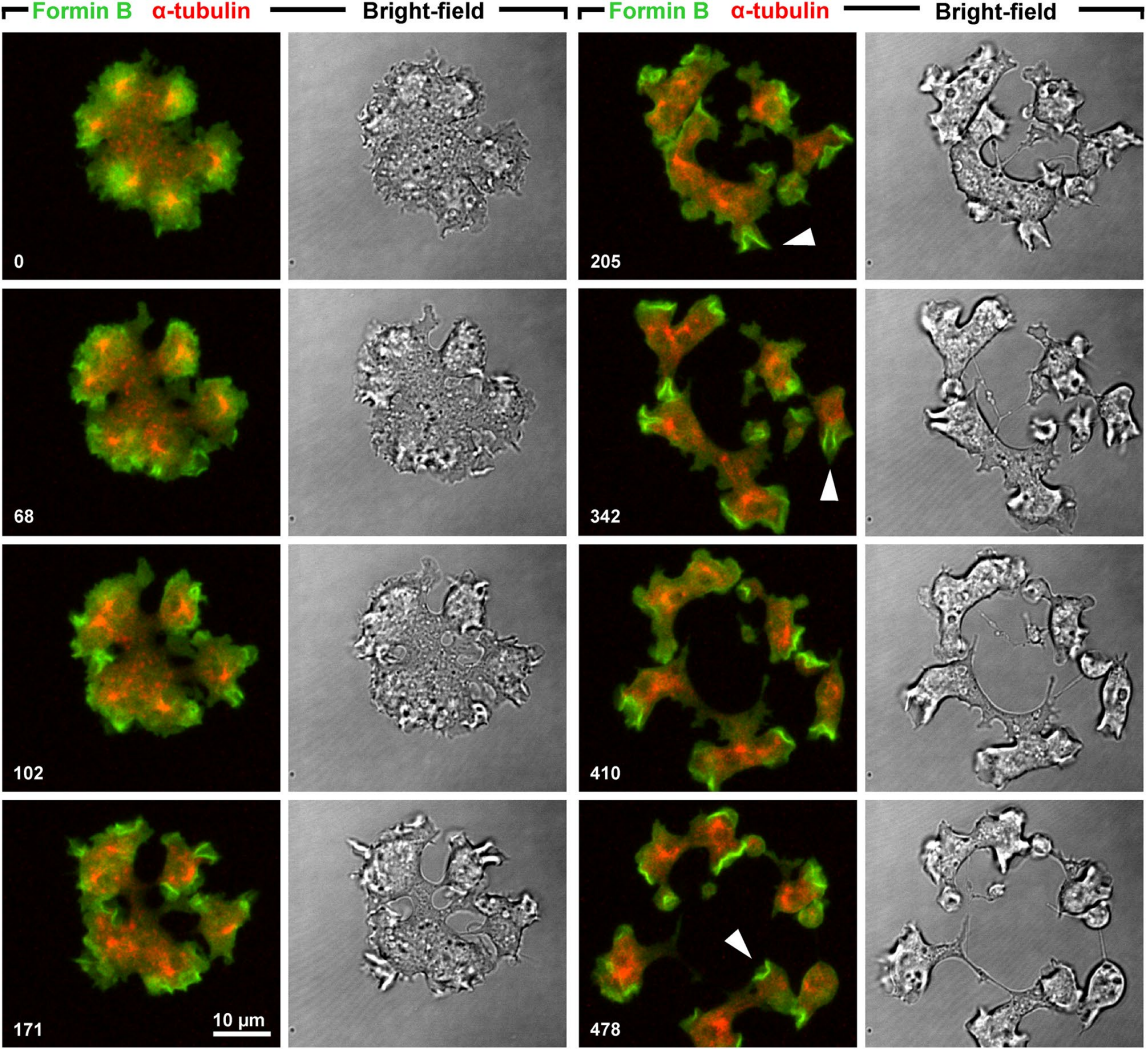
Division of a Septase-null cell by multiple unilateral cleavage furrows. The cell expressed GFP-ForBΔDAD (green) and RFP-α-tubulin (red). Time is indicated in seconds. Initially, ForB is accumulated in zones around the centrosomes, where aster microtubules contact the substrate-attached cell membrane. In the course of cleavage furrow progression, ForB redistributes to the fronts of daughter cells and to macropinocytic cups (arrowheads in 205-s, 342-s, and 478-s frames). This figure is related to Supplementary Video S2.

Figure 2 and Supplementary Video S2 show a cell that divided by multiple unilateral furrows. Again, at the beginning of furrow ingression ForB was enriched in the areas around centrosomes, a localization that subsequently got lost. In the daughter cells, ForB became enriched at the fronts and also in macropinocytic cups as recently reported by Körber et al.^10^.

**Figure 3:**
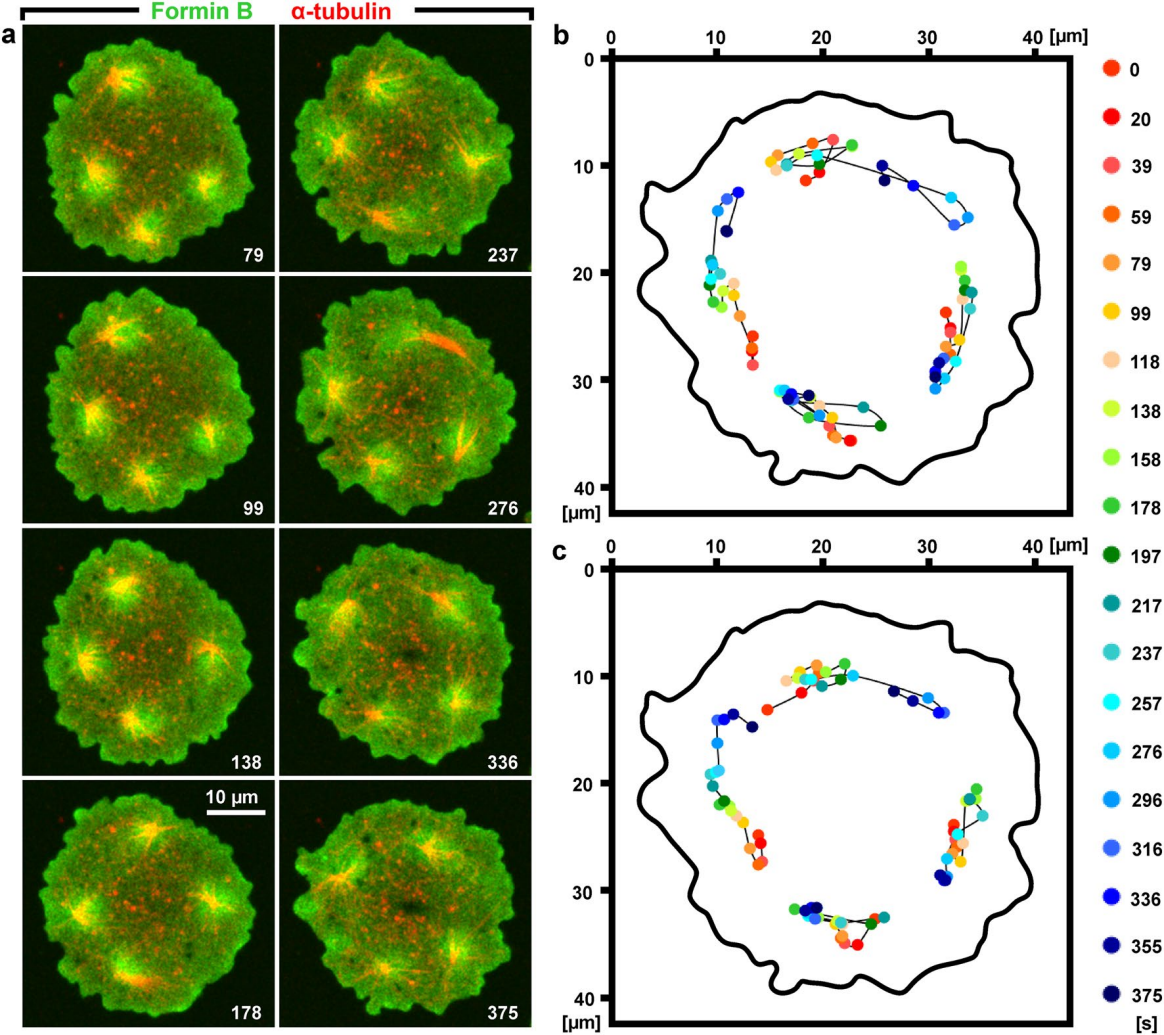
Centrosome movement followed by ForB accumulation. (**a**) Stages of repositioning of four centrosomes with associated microtubules (red), and changing areas of ForB accumulation (green). (**b**) Positions of the four centrosomes at time points color coded as in the scale on the right. (**c**) Positions of the geometric centers of ForB accumulations at time points corresponding to (**b**). Time is indicated in seconds consistently for (**a**–**c**). The full sequence of this recording is shown in Video S3.

After disassembly of the spindle, the centrosomes often changed their position, obviously driven by pulling force applied by dynein on microtubules^11,12^. In these cases, the ForB-decorated area followed the centrosome into its new position (Fig. 3 and Supplementary Videos S1 and S3), indicating that the ForB localization to the microtubule asters depends on signals that are continuously elicited during the period of ForB binding.

**Figure 4:**
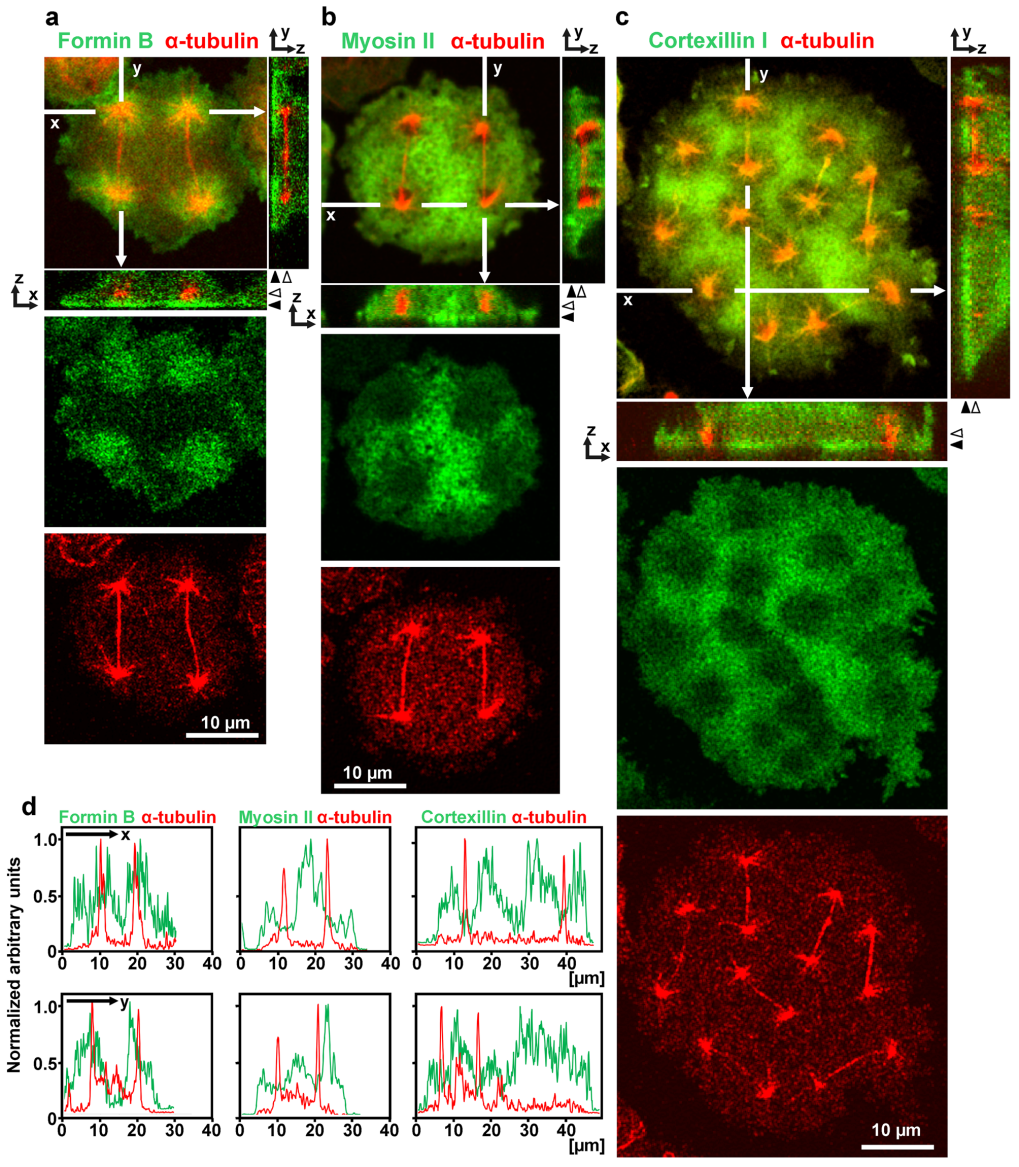
Accumulation of ForB and depletion of myosin II and cortexillin at the regions of microtubule asters. (**a**–**c**) Fluorescence images. The cell in (**a**) expressed GFP-ForBΔDAD, the cell in (**b**) GFP-myosin II heavy chain, and the cell in (**c**) GFP-cortexillin I (green). All cells expressed in addition RFP-α-tubulin as a label of the mitotic apparatus (red). The side-views show fluorescence intensities in z-direction. (**a**–**c**) show in the top panels average projections of the merged labels with the x- and y-axes indicating the position of the side views at the right and below the panels. Middle panels show fluorescence in a confocal plane close to the glass-attached cell surface as indicated in the side view by filled arrowheads. Bottom panels view the fluorescence of spindles and centrosomes at a plane indicated by open arrowheads. (**d**) Line scans of fluorescence intensities of 0.5 µm width along the x- and y-axes indicated in the top panels of (**a**–**c**) in the planes of the middle (green) or bottom panels (red).

The accumulation of ForB in the aster region contrasts to the depletion of myosin II and cortexillin in that region, indicating that two groups of actin—associated proteins are handled by sorting mechanisms that act in opposite directions. Figure 4 shows side-by-side the opposite sorting of ForB on one hand and myosin II and cortexillin on the other. For quantification, the confocal plane close to the glass-attached cell membrane was selected in which the contrast between aster areas and the areas between them was highest. Fluorescence intensities in this plane determined in line scans of 0.5 µm width along the x- and y-axes are shown in Fig. 4d.

### Mobile ForB patches on the substrate-attached cell membrane

**Figure 5:**
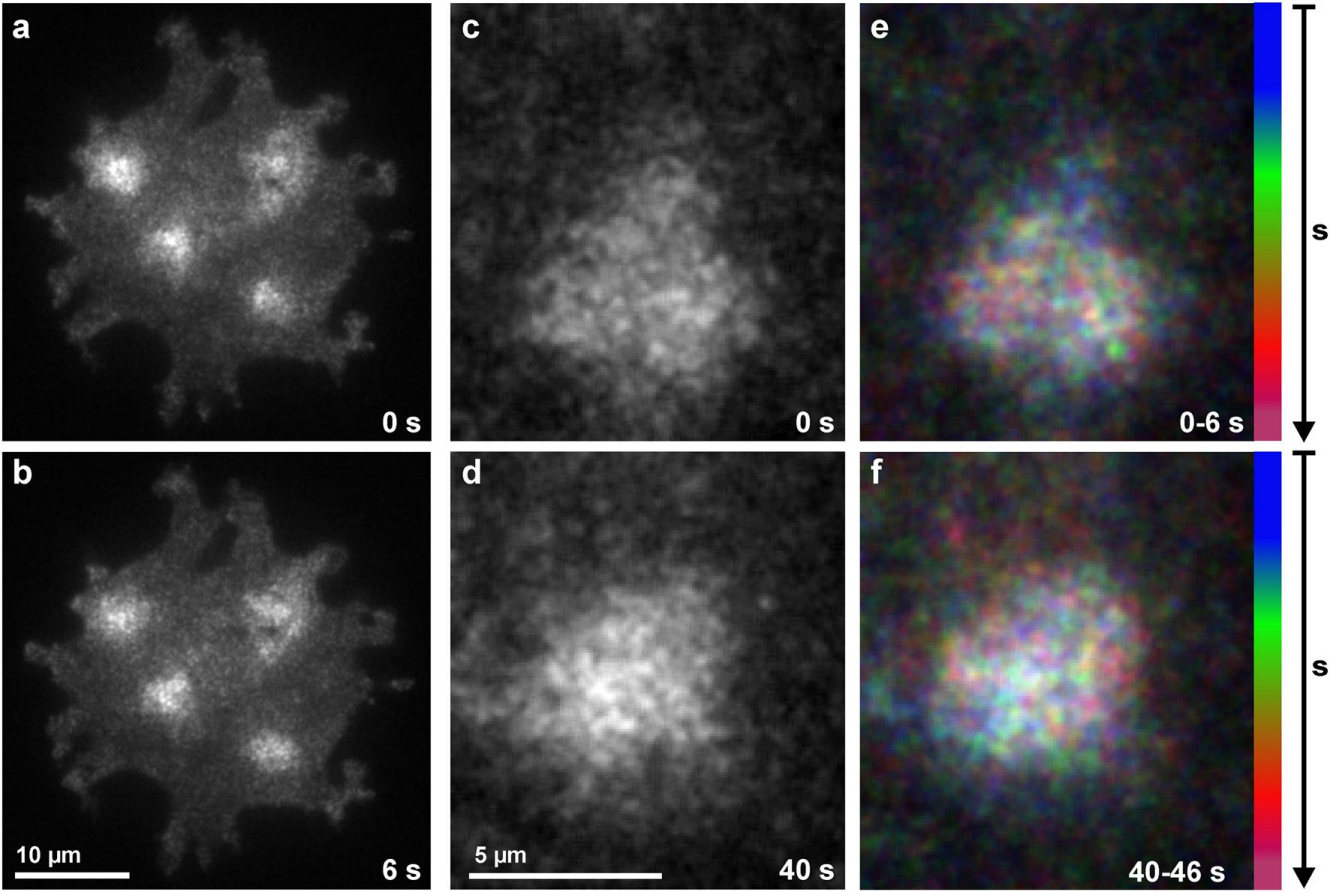
Mobile ForB patches on the substrate-attached membrane as revealed by TIRF microscopy. Dividing SepA-null cells that expressed GFP-ForBΔDAD were imaged in TIRF mode to visualize structures close to the substrate surface. (**a**,**b**) Cell with four ForB-enriched areas. (**c**,**d**) A single ForB-enriched area in another cell showing two TIRF recordings taken at a 40-s interval. (**e**,**f**) Temporal color-coded fluorescence intensities in five consecutive frames taken during the period of 0–6 s (**e**) or 40–46 s (**f**) to demonstrate motility of the ForB patches.

To specify the location of ForB that is enriched in the areas of the microtubule asters, we applied total internal reflection fluorescence (TIRF) microscopy, which selectively visualizes fluorescent structures within the evanescent field above the reflecting substrate surface. In the aster regions, ForB is visualized by TIRF in the form of patches that are mobile in the plane of the membrane (Fig. 5). This result suggests that it is a microtubule-dependent modification of membrane composition that enables the ForB to bind.

### Dynamics of actin and Arp2/3 clustering

**Figure 6:**
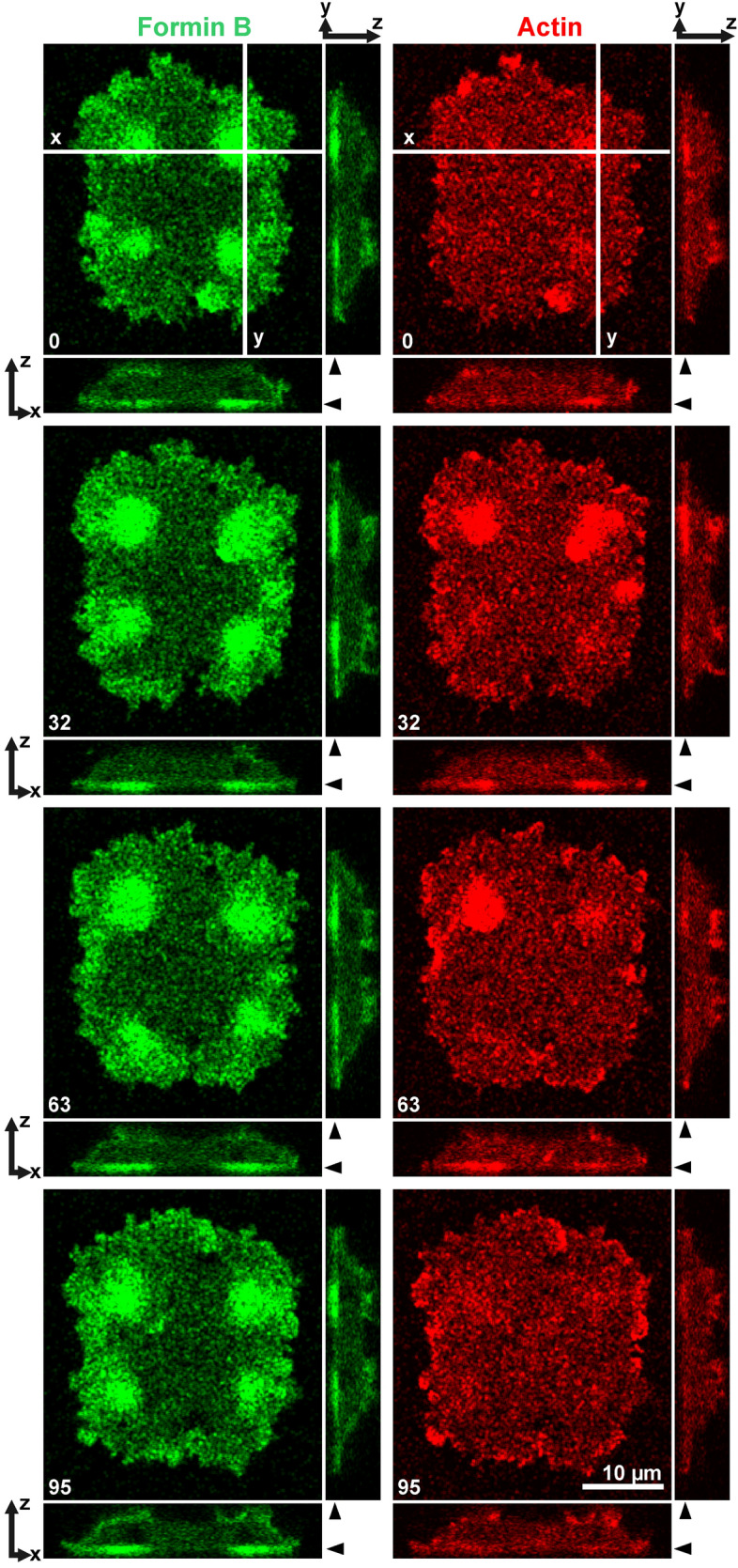
Localization of ForB and actin in late anaphase to microtubule asters that surround the centrosomes. The cell expressed GFP-ForBΔDAD (green) and mRFP-LimEΔ as a label for filamentous actin (red). The two labels are shown in a confocal xy-plane close to the substrate-attached cell surface, and on the bottom and right of these panels in z-direction along the x- and y-axes as indicated in the 0-s image. Arrowheads point to z-position of the confocal xy-plane. The cell subsequently divided into four cells (not shown). Time is indicated in seconds.

Comparison of a label for filamentous actin with the GFP-ForB construct revealed that the actin label also accumulated in the region of microtubule asters. However, the ForB label more uniformly covered a circular area around each centrosome, whereas the actin label showed short-lived clusters of irregular shape and size (Fig. 6).

**Figure 7:**
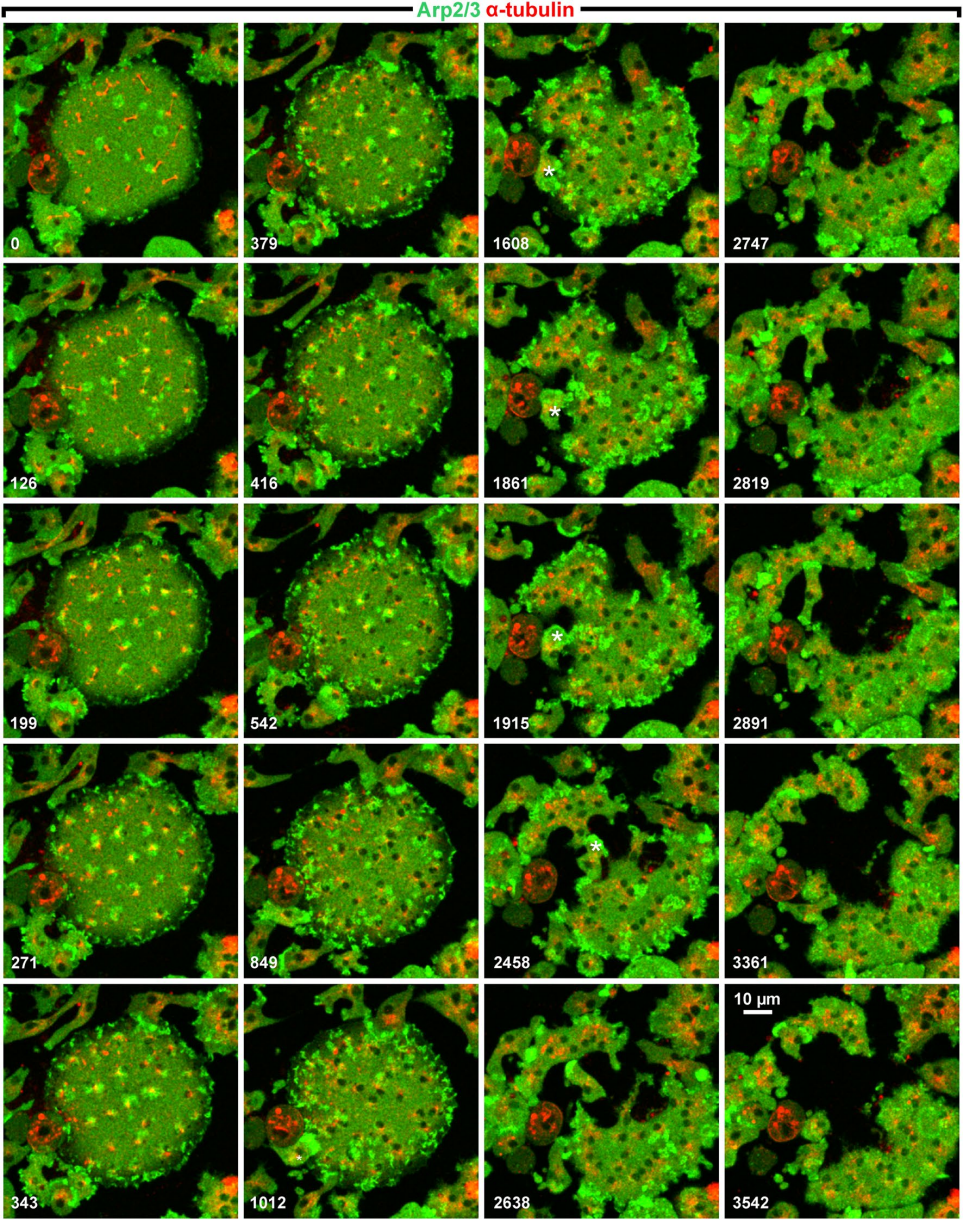
Changing localizations of Arp2/3 in a large multinucleate cell. The cell expressed GFP-Arp3 (green) and RFP-α-tubulin (red). The GFP-Arp3 clustered at the beginning independently of the positions of microtubule complexes (0-s to 126-s frames). During a period from the 199-s up to the 343-s frames, clusters became associated with the regions around centrosomes. Finally, the Arp2/3 label was incorporated into propagating waves, visible at the lower right corner of the images. An interphase cell that penetrates into the mitotic one and assists in its cleavage is marked by asterisks in the 1012-s to 2458-s frames. Time is indicated in seconds. This figure is related to Supplementary Video S4.

Redistribution of the Arp2/3 complex during mitosis of a multinucleate cell was monitored using GFP-Arp3 as a label. Figure 7 and Supplementary Video S4 cover all phases of the mitotic cycle, and in all of them the Arp2/3 complex was in one or the other way clustered. The sequence begins with an early anaphase stage in which the spindles were not yet fully elongated. At this stage, GFP-Arp3 was found in short-lived clusters distributed on the cell area independently of the microtubule system. These clusters probably represented rudimentary actin waves, as they are seen fully developed in the interphase cell on the upper right corner of the images. During a short period of late anaphase, GFP-Arp3 clustered around the centrosomes in structures of irregular and continuously changing shape (199-s to 343-s frames). Subsequently, clustering became again independent of the microtubule complexes and was prominent at the cell border (1012-s to 1915-s frames). Finally, propagating waves appeared, as they are typical of interphase cells (2458-s to 3542-s frames)^13^.

A behavior of interphase cells seen in Fig. 7 and Supplementary Video S4 should be mentioned since it is a common feature in *Dictyostelium*: midwifing as it has been called by Biron et al.^14^. Here an interphase cell, marked by an asterisk in Fig. 7 (1608-s to 2458-s frames), penetrated into the mitotic cell and cleaved this cell along its path (see Supplementary Video S4 for details).

### Ingressing unilateral furrows

**Figure 8:**
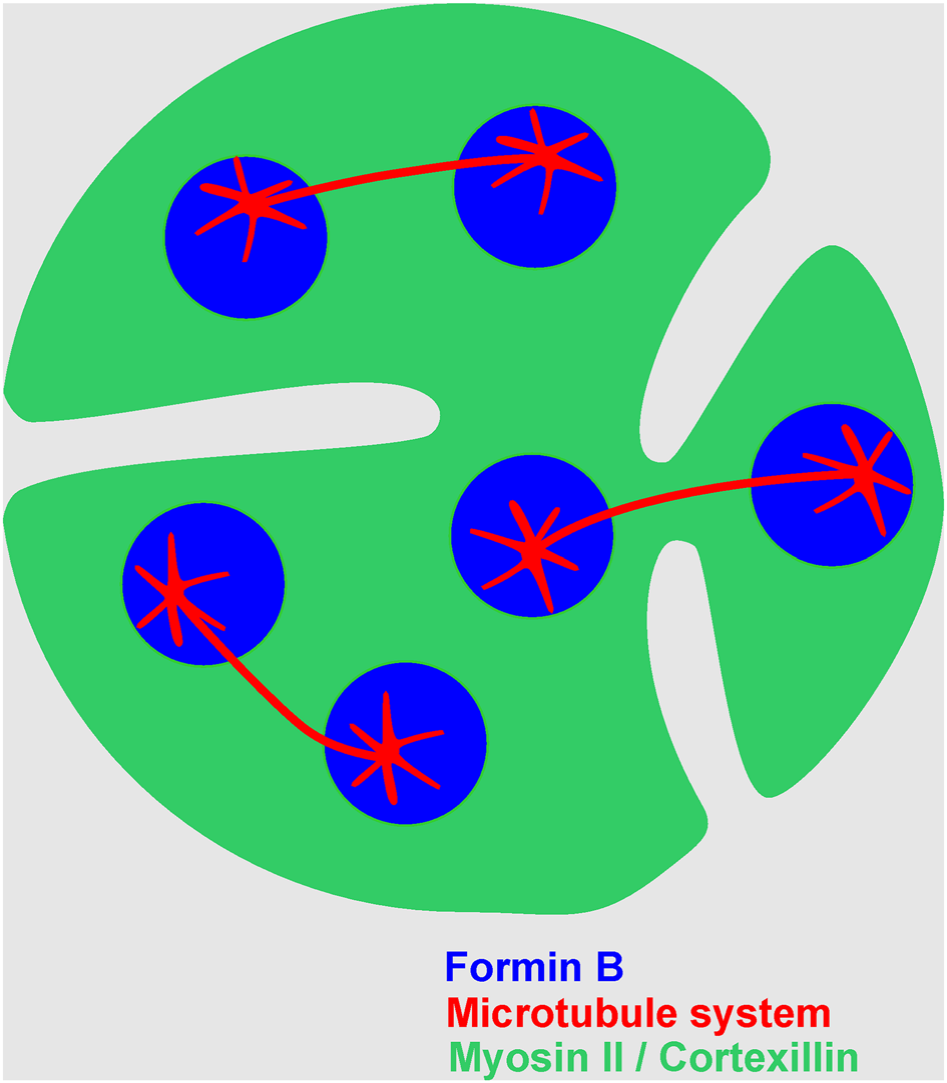
Scheme of microtubule-dependent patterns in the substrate-attached cortex of a multinucleate cell in mitosis. The microtubule system is shown in red, ForB-covered areas in blue, and areas enriched in myosin II and cortexillin in green. During the anaphase of mitosis, the region of astral microtubules around the centrosomes is distinguished by the recruitment of ForB and the displacement of myosin II and cortexillin. Unilateral cleavage furrows that ingress from the cell border avoid the aster regions. Furrows that meet will cleave off a daughter cell, as supposed for the two furrows on the right. The figure compiles data published previously (Gerisch et al.) and results reported in the present paper.

Previous work has shown that in multinucleate cells, cleavage furrows ingress from the cell border along paths that are rich in myosin II and cortexillin, and are free of microtubule asters^3^ (Fig. 8). At the furrows, we did neither find ForB (Figs. 1 and 2) nor Arp2/3 (Fig. 7) to accumulate. This finding is in accord with the localization of Arp2/3 in normally dividing cells, where this complex accumulates in polar protrusions rather than in the cleavage furrows^15^.

## Discussion

In this report we addressed the localization of Formin B and Arp2/3, both involved in actin polymerization, during the mitosis of multinucleate cells in a SepA-mutant of *Dictyostelium discoideum,* which lack a serine-threonine kinase. Cells of this mutant do not round up during mitosis. In the SepA-null cells, a planar pattern is induced by the microtubule asters. In this pattern on the cytoplasmic face of the substrate-attached membrane, ForB/Arp2/3 alternate with myosin II / cortexillin (Figs. 4 and 8). The patterning of the cell cortex by microtubule asters also involves the positioning of unilateral cleavage furrows, which circumvent the aster regions on their path into the cell^3^. This positioning is peculiar, as sites for furrow ingression are preferred where the centrosomes at the spindle poles have the largest distance from each other. This is the opposite of what one would expect if cleavage furrows are determined at sites of highest density of aster microtubules^16^. The presence of a spindle is not required for furrow ingression^11^. Our findings are in accord with the discussion by Okada and Yumura^17^ on cleavage furrow positioning in *Dictyostelium*.

During anaphase, Formin B showed a prominent localization to the substrate-attached cell surface within the zones of microtubule asters that radiated out of the centrosomes. In the course of cytokinesis, the localization of ForB became restricted to protrusions and invaginations of the cell border. The question is whether the re-localization of ForB involves a change in binding sites. Körber et al.^10^ identified RacB as a major binding partner of ForB in *Dictyostelium* membranes, but we know that ForB still binds in RacB-null cells^18^. A player to be considered in specifying a cleavage furrow is Rap1, which inhibits the assembly of myosin II at the cell cortex^19^. In the multinucleate cells studied, the region of microtubule asters, where ForB and Arp2/3 assemble, is the one that is spared off by myosin-II and cortexillin^3^.

The GFP-ForB construct used proved to be the most distinct marker of the areas around centrosomes, in comparison to the actin or Arp2/3 labels. The localization of ForB resembles the rings built by the formin FMN1 in cytotoxic T cells. In these cells the formin is involved in reorientation of the centrosome toward the immunological synapse formed in response to contact with an antigen presenting cell^20^.

In *Dictyostelium*, the ForB-enriched areas associated with microtubule asters are related to MiDAses, mitosis-specific dynamic actin structures on the ventral, substrate-attached surface of myosin II-null cells reported by Itoh and Yumura^21^ to be formed in regions controlled by microtubule asters. MiDASes proved to be areas of strong adhesion of the cells to the substrate that were required for the cells to divide in the absence of myosin II. The Arp2/3 complex and its organizer WASP have been shown by King et al.^15^ to localize to MiDASes and to be essential for their function.

We wish to underscore that the Arp2/3 complex formed clusters on the substrate-attached cell area all the time during mitosis, but only during a short period of late anaphase the complex clustered around the centrosomes (Fig. 7). An additional feature illustrated in Fig. 7 is the cessation of wave propagation during mitosis, indicating that in *Dictyostelium* cells the excitability of the actin cortex is altered during cell division.

## Conclusion

We apply multinucleate cells of a *Dictyostelium* mutant that are spread on a substratum and generate planar patterns of proteins that in normal mitosis are either localized to the cleavage furrow or to polar protrusions of the dividing cell. In accord with the pattern that is induced by microtubule asters, the cell is cleaved by unilateral furrows. Our data illustrate a microtubule-dependent sorting mechanism for actin-binding proteins that is activated at anaphase.

## Material and methods

### Cell strains

Septase-null mutants^22^ derived from the AX2-214 strain of *Dictyostelium discoideu*m that expressed green and red fluorescent proteins (GFP and RFP, respectively) were used for all experiments (see Supplementary Table S1). The vectors used to express the fluorescent proteins enable constitutive expression of the fluorescent fusion proteins in *Dictyostelium*, and are randomly integrated into the genome. They are introduced to the cells by electroporation and maintained by antibiotic selection^23^. The ForB probe used has been a ΔDAD construct lacking the inhibitory domain as described by Körber, et al.^10^.

### Culture conditions and sample preparation for microscopy

Cells were cultivated in nutrient medium as described by Malchow et al.^24^, supplemented with 10 µg/ml of Blasticidin S (B10) (Gibco, Life Technologies Corporation, Grand Island, NY, USA), 10 µg/ml of Geneticin (G10) (Sigma-Aldrich, St. Louis, MO, USA), and/or of 33 µg/ml of Hygromycin B (H33) (Calbiochem, Merck KGaA, Darmstadt, Germany) in plastic Petri dishes at 21 ± 2 °C.

To increase the rate of mitotic stages in the multinucleate Septase-null cells, the nutrient medium was removed and the cells were rinsed off the Petri dishes with Loflo medium (ForMedium Ltd., Norfolk, UK). The cells were then transferred to HCl-cleaned cover-glass bottom dishes (FluoroDish, WPI INC., Sarasota, FL, USA) and either incubated overnight at 21 ± 2 °C in Loflo medium or imaged the same day after incubation for 1–2 h in Loflo medium. Before imaging, cells were overlaid by a thin agarose sheet^25^ that had been incubated in Loflo medium.

### Image acquisition and data processing

A Zeiss LSM 780 inverted microscope equipped with a Plan-Apochromat 63x/NA 1.46 oil objective (Zeiss AG, Oberkochen, Germany) was used to image confocal planes with a z-distance of 0.15 µm. TIRF images of the substrate-attached membrane region were acquired by illumination through a high-aperture objective^26^, a OLYMPUS UAPON TIRF 100X/1.49 oil objective (Evident Scientific, Inc., Waltham, USA), at a GE DeltaVision Elite system (GE Healthcare Bio-Sciences Corp., Marlborough, MA, USA) based on an OLYMPUS IX-71 inverted microscope, a PCO sCMOS 5.5 camera and a TIRF/PK module controlled by the softWoRx® program, version 7. To excite the GFP-ForBΔDAD in the cells we used the 488-nm laser (100 mW) and for detection a 525/48 emission filter. To ensure stringent TIRF conditions, after focusing in widefield mode on the glass attached part of the cells, we carefully adjusted in TIRF mode the angle of the 488-nm diode laser with the TIRF illumination settings of the Delta Vision Imaging System by dragging the box in the slider bar from epifluorescence to TIRF. Then we made a fine adjustment to the TIRF angle by clicking the arrows of the slider bar until we saw only background and after that going back just until we saw fluorescent signals. The finding of the angle is supported by the instrument program where we can see that we are in the TIRF marked area.

The refractive index for *Dictyostelium* is assumed to vary between n = 1.368 and 1.373^27^. According to the producers’ datasheet, the refractive index for the glass used for Fluorodishes (0.17 ± 0.01 mm, WPI INC., Sarasota, FL, USA) is n = 1.525. These are the conditions under which according to Axelrod^26^ the TIRF recordings are based.

The images were processed using the image-processing package Fiji (http://Fiji.sc/Fiji) developed by Schindelin et al.^28^ on the basis of ImageJ (http://imagej.nih.gov/ij). If not indicated otherwise, we show average projections of series of confocal z-planes. Because the RFP label tended to bleach at long periods of imaging, we corrected the signal in the red channel with the “Bleach Correction” plugin in “Simple Ratio Mode”. For tracking the centrosomes and ForB accumulations in Fig. 3b,c the “Manual Tracking” tool was employed. For the ForB accumulations, the images were binarized, contours of the accumulations were evaluated and the geometric centers were calculated with the ImageJ “Centroid” function. These centers were used for tracking. Line scans with a width of 0.5 µm were made with the “Straight Line” tool (Fig. 4d). The “Orthogonal Views” tool was used in Figs. 4a–c and 6 to show the 3D distribution of fluorescent proteins in xz- and yz-direction. To analyze the motile ForB patches in the TIRF mode a “Temporal-color code” tool with a modified rainbow LUT was applied for 5 time points in succession in Fig. 5e,f.

### Supplementary Information

Supplementary Information 1.

Supplementary Video 1.

Supplementary Video 2.

Supplementary Video 3.

Supplementary Video 4.

## Data Availability

Original data is available upon request from the corresponding author.

## References

[1] Burgess DR, Chang F (2005). Site selection for the cleavage furrow at cytokinesis. Trends Cell Biol..

[2] Ecke, M., Prassler, J. & Gerisch, G. Expanding ring-shaped cleavage furrows in multinucleate cells. *Mol. Biol. Cell* **34**, ar27. 10.1091/mbc.E22-10-0487 (2023). 10.1091/mbc.E22-10-0487 PMC10092652 36652336

[3] Gerisch, G., Prassler, J. & Ecke, M. Patterning of the cell cortex and the localization of cleavage furrows in multi-nucleate cells. *J. Cell Sci.* **135**, jcs259648. 10.1242/jcs.259648 (2022). 10.1242/jcs.259648 PMC9016623 35274133

[4] Bindl, J. *et al.* Unilateral cleavage furrows in multinucleate cells. *Cells* **9**. 10.3390/cells9061493 (2020). 10.3390/cells9061493 PMC7349700 32570994

[5] Fukui Y, Inoué S (1991). Cell division in dictyostelium with special emphasis on actomyosin organization in cytokinesis. Cell Motil. Cytoskeleton.

[6] Weber I (1999). Cytokinesis mediated through the recruitment of cortexillins into the cleavage furrow. EMBO J..

[7] Yumura S, Mori H, Fukui Y (1984). Localization of actin and myosin for the study of ameboid movement in dictyostelium using improved immunofluorescence. J. Cell Biol..

[8] Insall R (2001). Dynamics of the dictyostelium Arp2/3 complex in endocytosis, cytokinesis, and chemotaxis. Cell Motil..

[9] Ecke M (2020). Formins specify membrane patterns generated by propagating actin waves. Mol. Biol. Cell.

[10] Körber S, Junemann A, Litschko C, Winterhoff M, Faix J (2023). Convergence of Ras- and Rac-regulated formin pathways is pivotal for phagosome formation and particle uptake in dictyostelium. Proc. Natl. Acad. Sci. USA.

[11] Neujahr R (1998). Microtubule-mediated centrosome motility and the positioning of cleavage furrows in multinucleate Mosin II-null cells. J. Cell Sci..

[12] Koonce MP (1999). Dynein motor regulation stabilizes interphase microtubule arrays and determines centrosome position. EMBO J..

[13] Bretschneider T (2004). Dynamic actin patterns and Arp2/3 assembly at the substrate-attached surface of motile cells. Curr. Biol..

[14] Biron D, Libros P, Sagi D, Mirelman D, Moses E (2001). A sexual reproduction: 'Midwives' assist dividing amoebae. Nature.

[15] King JS, Veltman DM, Georgiou M, Baum B, Insall RH (2010). SCAR/WAVE is activated at mitosis and drives myosin-independent cytokinesis. J. Cell Sci..

[16] Devore JJ, Conrad GW, Rappaport R (1989). A model for astral stimulation of cytokinesis in animal cells. J. Cell Biol..

[17] Okada A, Yumura S (2023). Cleavage furrow positioning in dividing Dictyostelium cells. Cytoskeleton.

[18] Ecke M, Prassler J, Gerisch G (2023). Fluctuations of formin binding in the generation of membrane patterns. Biophys. J..

[19] Plak K, Keizer-Gunnink I, van Haastert PJM, Kortholt A (2014). Rap1-dependent pathways coordinate cytokinesis in Dictyostelium. Mol. Biol. Cell.

[20] Gomez TS (2007). Formins regulate the actin-related protein 2/3 complex-independent polarization of the centrosome to the immunological synapse. Immunity.

[21] Itoh G, Yumura S (2007). A novel mitosis-specific dynamic actin structure in Dictyostelium cells. J. Cell Sci..

[22] Müller-Taubenberger A, Ishikawa-Ankerhold HC, Kastner PM, Burghardt E, Gerisch G (2009). The STE group kinase SepA controls cleavage furrow formation in Dictyostelium. Cell Motil. Cytoskeleton.

[23] Eichinger, L. & Rivero, F. *Methods in Molecular Biology—Dictyostelium discoideum Protocols*. Vol. 346 (Chapter 11, 14) (Humana Press, 2006).

[24] Malchow D, Nägele B, Schwarz H, Gerisch G (1972). Membrane-bound cyclic AMP phosphodiesterase in chemotactically responding cells of Dictyostelium discoideum. Eur. J. Biochem..

[25] Fukui, Y., Yumura, S. & Yumura, T. K. in *Methods Cell Biol* Vol. 28 (ed James A. Spudich) 347–356 (Academic Press, 1987). 10.1016/s0091-679x(08)61655-6 3298995

[26] Axelrod D (2001). Total internal reflection fluorescence microscopy in cell biology. Traffic.

[27] Gingell D, Vince S (1982). Substratum wettability and charge influence the spreading of Dictyostelium Amoebae and the formation of ultrathin cytoplasmic lamellae. J. Cell Sci..

[28] Schindelin J (2012). Fiji: An Open-Source Platform for Biological-Image Analysis. Nat Meth.

