# Supplementary material for: Microtubule dependent sorting of actin-binding proteins in mitosis
Source: Sci Rep. 2024 May 16;14:11250. doi: 10.1038/s41598-024-61967-7 (PMC11099102; doi:10.1038/s41598-024-61967-7)
Supplement: Supplementary file 1 — Supplementary Information 1. [file 41598_2024_61967_MOESM1_ESM.pdf]

Microtubule dependent sorting of actin-binding proteins in mitosis

Jana Prassler[1], Mary Ecke[2], Günther Gerisch[3*]

[1, 2, 3] Max Planck Institute of Biochemistry, Am Klopferspitz 18, 82152 Martinsried, Germany

*Corresponding author: gerisch@biochem.mpg.de

Supplemental Video Legends, Table and References

Supplementary Video Legends

Video S1. This video of a cell that divided by ingression of a single furrow shows the same recording as Figure 1. The cell expressed GFP-ForBΔDAD (green) and RFP-α-tubulin (red). In the first six frames (displayed slowly) the translocation of the ForB-rich areas together with the moving centrosomes is recognizable.

Video S2. This video shows the division by multiple unilateral furrows of a cell that expressed GFP-ForBΔDAD (green) and RFP-α-tubulin (red). It is related to Figure 2. During late anaphase the ForB label accumulated in areas surrounding the centrosomes at the ends of the elongated spindles.

Video S3. This video shows the movement of four centrosomes (red) followed by the translocation of ForB-decorated areas (green). Selected frames and two diagrams of the same recording are shown in Figure 3.

Video S4. This video shows the same recording as Figure 7 of a large multinucleate cell that expressed GFP-Arp3 (green) and RFP-α-tubulin (red). The sequence illustrates localization of GFP-Arp3 close to the centrosomes during the late anaphase stage (period from 18-s to 1012-s). Upon transition of daughter cells to the interphase, the label became incorporated into propagating waves (at the lower right corner beginning at 2638-s). In the 1012-s to 2476-s frames a small interphase cell is seen to assist the large cell in cleaving.

Supplementary Table S1. Strains

Strain	GFP Label	RFP Label	Resistance	References
Septase-null	pDGFP-ForBΔDAD[a]	pDRH-Hyg^R:RFP-α-tubulin	G10 / H33[b]	[1-3]
Septase-null	pDGFP-ForBΔDAD[a]	mRFPM-LimEΔ	G10 / B10[c]	[1,3,4]
Septase-null	pDRH-HygR:RFP-α-tubulin	GFP-Arp3	G10 / H33[b]	[2,5]
Septase-null	pDRH-HygR:RFP-α-tubulin	GFP-myosin II	G10 / H33[b]	[2,6]
Septase-null	pDRH-HygR:RFP-α-tubulin	GFP-cortexillin I	G10 / H33[b]	[2,7]

[a] ΔDAD = diaphanous autoinhibitory domain deleted
[b] G10 = 10 µg / ml Geneticin / H33 = 33µg / ml Hygromycin B
[c] G10 = 10 µg / ml Geneticin / B10 = 10 µg / ml Blasticidin S

References for GFP or RFP Label in Supplementary Table S1

1 Ecke, M. *et al.* Formins Specify Membrane Patterns Generated by Propagating Actin Waves. *Mol Biol Cell* **31**, 373-385, doi:10.1091/mbc.E19-08-0460 (2020).

2 Effler, J. C. *et al.* Mitosis-specific Mechanosensing and Contractile-protein Redistribution Control Cell Shape. *Curr. Biol.* **16**, 1962-1967, doi:doi.org/10.1016/j.cub.2006.08.027 (2006).

3 Körber, S., Junemann, A., Litschko, C., Winterhoff, M. & Faix, J. Convergence of Ras- and Rac-Regulated Formin Pathways is Pivotal for Phagosome Formation and Particle Uptake in Dictyostelium. *Proc. Natl. Acad. Sci. USA* **120**, e2220825120, doi:10.1073/pnas.2220825120 (2023).

4 Fischer, M., Haase, I., Simmeth, E., Gerisch, G. & Müller-Taubenberger, A. A Brilliant Monomeric Red Fluorescent Protein to Visualize Cytoskeleton Dynamics in Dictyostelium. *FEBS Lett.* **577**, 227-232, doi:10.1016/j.febslet.2004.09.084 (2004).

5 Insall, R. *et al.* Dynamics of the Dictyostelium Arp2/3 Complex in Endocytosis, Cytokinesis, and Chemotaxis. *Cell Motility* **50**, 115-128, doi:doi.org/10.1002/cm.10005 (2001).

6 Robinson, D. N., Cavet, G., Warrick, H. M. & Spudich, J. A. Quantitation of the Distribution and Flux of Myosin-II During Cytokinesis. *BMC Cell Biol.* **3**, 4, doi:10.1186/1471-2121-3-4 (2002).

7 Weber, I. *et al.* Cytokinesis Mediated Through the Recruitment of Cortexillins into the Cleavage Furrow. *EMBO J.* **18**, 586-594, doi:10.1093/emboj/18.3.586 (1999).
